# Rare case of a giant thrombosed left anterior descending coronary artery aneurysm

**DOI:** 10.1186/s13019-020-01250-8

**Published:** 2020-07-30

**Authors:** Yong Peng, Yaxiong Li, Yu Jiang

**Affiliations:** 1 Department of Cardiovascular Sugary, Yan’an Hospital affiliated with Kunming Medical University, Kunming, Yunnan P.R. China; 2 Key Laboratory of Cardiovascular Disease, Kunming, Yunnan P.R. China

**Keywords:** Atherosclerosis, Coronary angiography, Coronary artery disease, Coronary artery aneurysm, Coronary artery bypass graft

## Abstract

**Background:**

Coronary artery aneurysms (CAAs) are rare, and giant CAAs are even rarer. The pathophysiology of this phenomenon is still unknown.

**Case presentation:**

Herein, we present the case of a 49-year-old male with a giant aneurysm in the left anterior descending artery.

**Conclusions:**

The optimal treatment for CAAs is debatable, but surgical intervention is preferred for giant CAAs.

## Background

Coronary artery aneurysms (CAAs) are rarely reported life-threatening abnormalities of the cardiovascular system [1]. CAAs were first described by Morgagni in 1761 [2]. A CAA is defined as a dilation of the normal coronary diameter to 1.5 times the size of a normal adjacent coronary artery segment [3]. If the diameter of a CAA is greater than four times the normal size or > 8 mm, it is considered a giant CAA [4]. Aneurysms are often found by coronary angiography, with an incidence ranging from 0.1 to 4.9%. The right coronary artery (RCA) is most commonly affected, followed by the left circumflex (LCX) artery and the left anterior descending (LAD) artery. Although rare, an aneurysm could undergo thrombosis and spontaneous dissection, leading to acute myocardial infarction (MI) [5].

Herein, we present the case of a 49-year-old male with a giant CAA in the LAD artery.

**Fig. 1:**
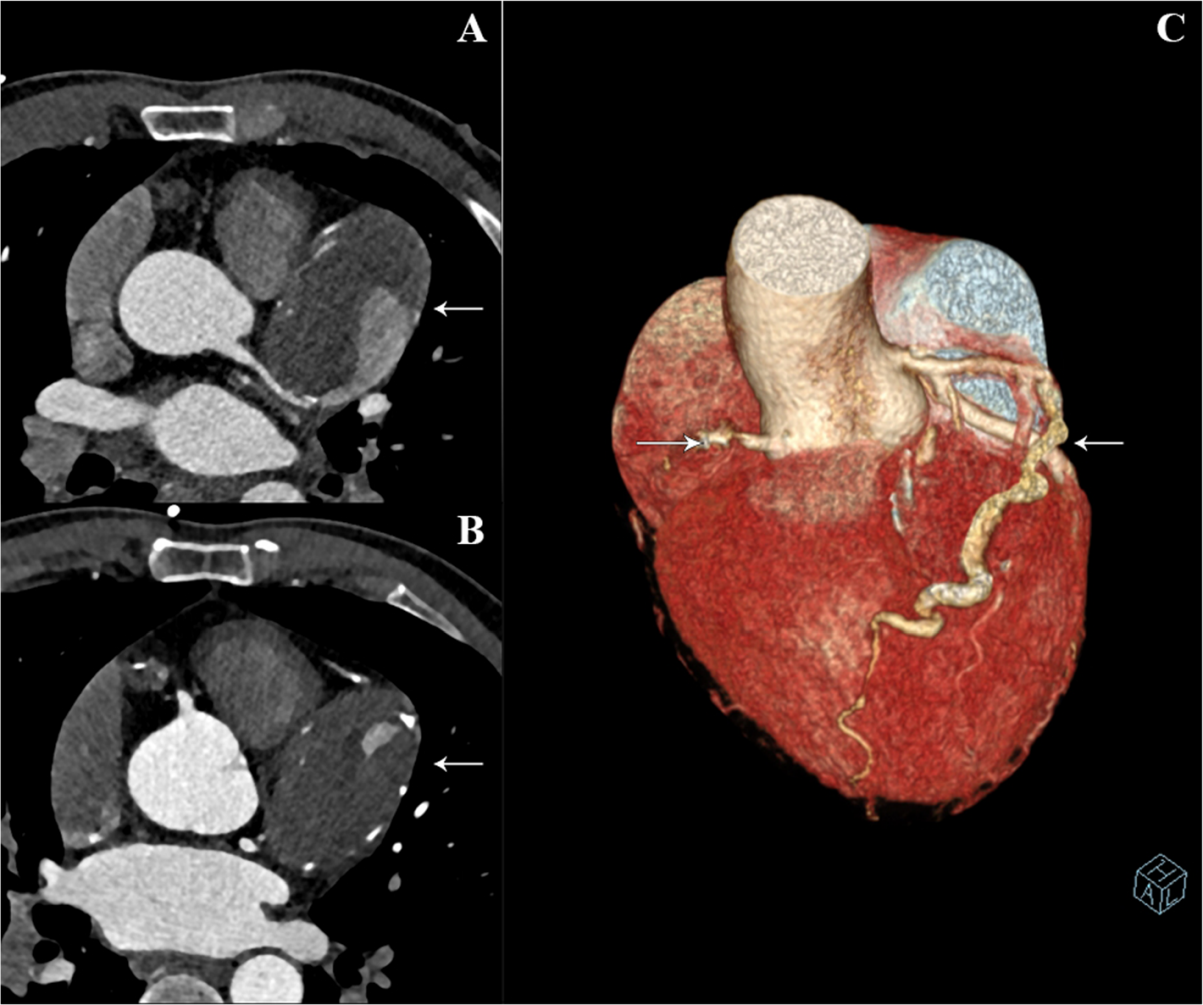
Dual-source computed tomography showing the giant aneurysm and the affected vessel

**Fig. 2:**
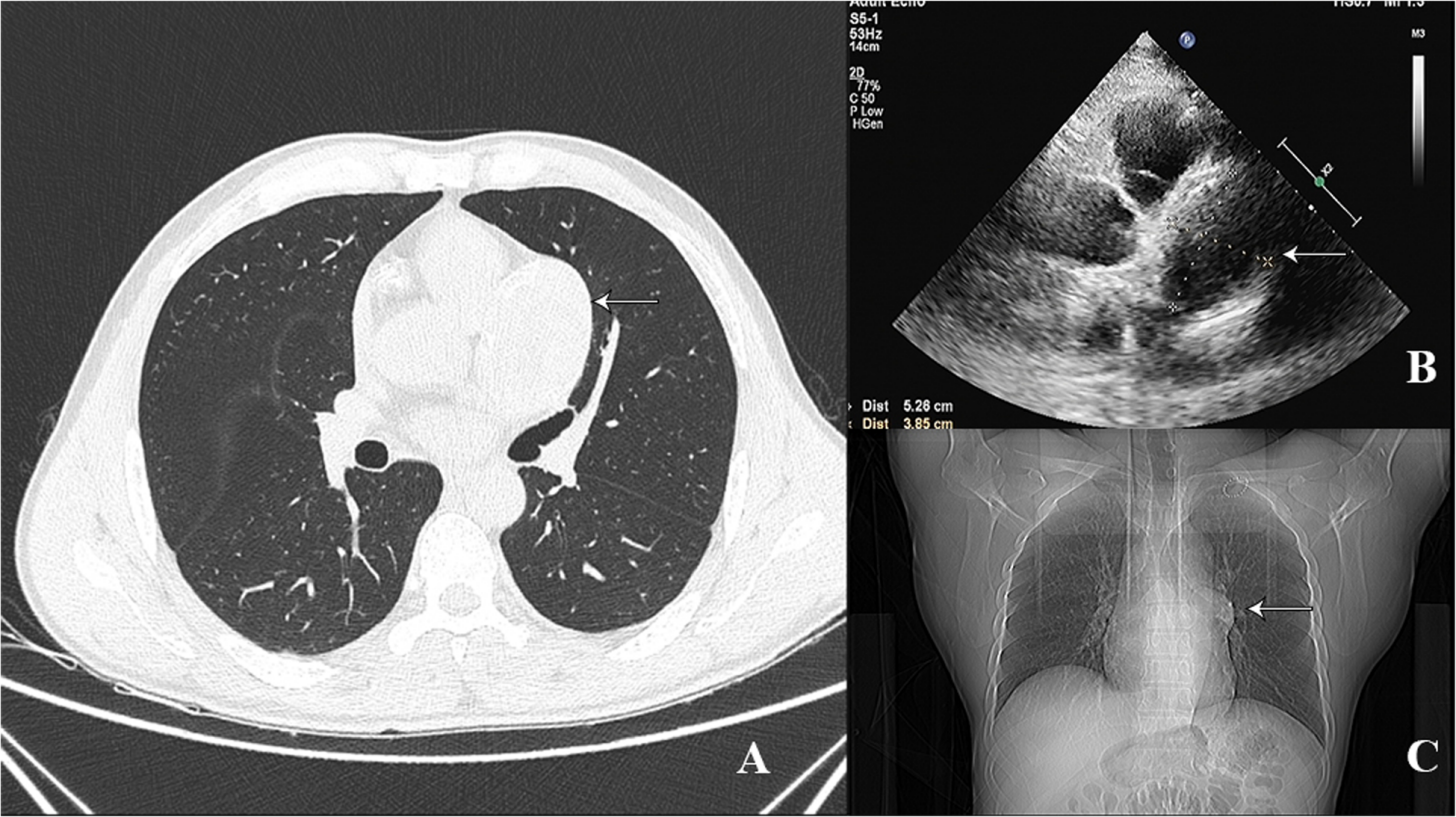
Computed tomography, echocardiography and X-ray examination revealing the aneurysm (arrows)

**Fig. 3:**
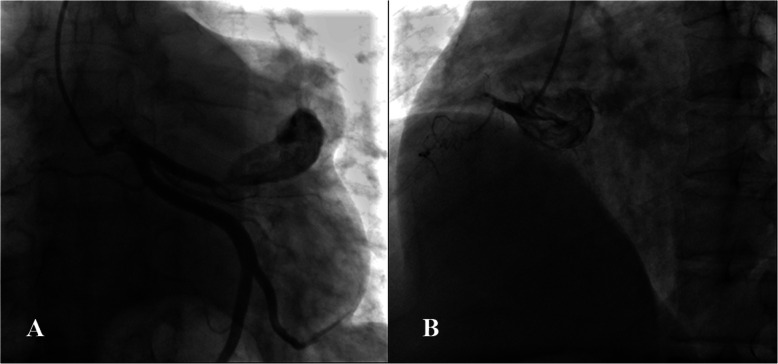
Coronary angiography demonstrating the aneurysm in the proximal left anterior descending artery and occlusion of the right coronary artery

**Fig. 4:**
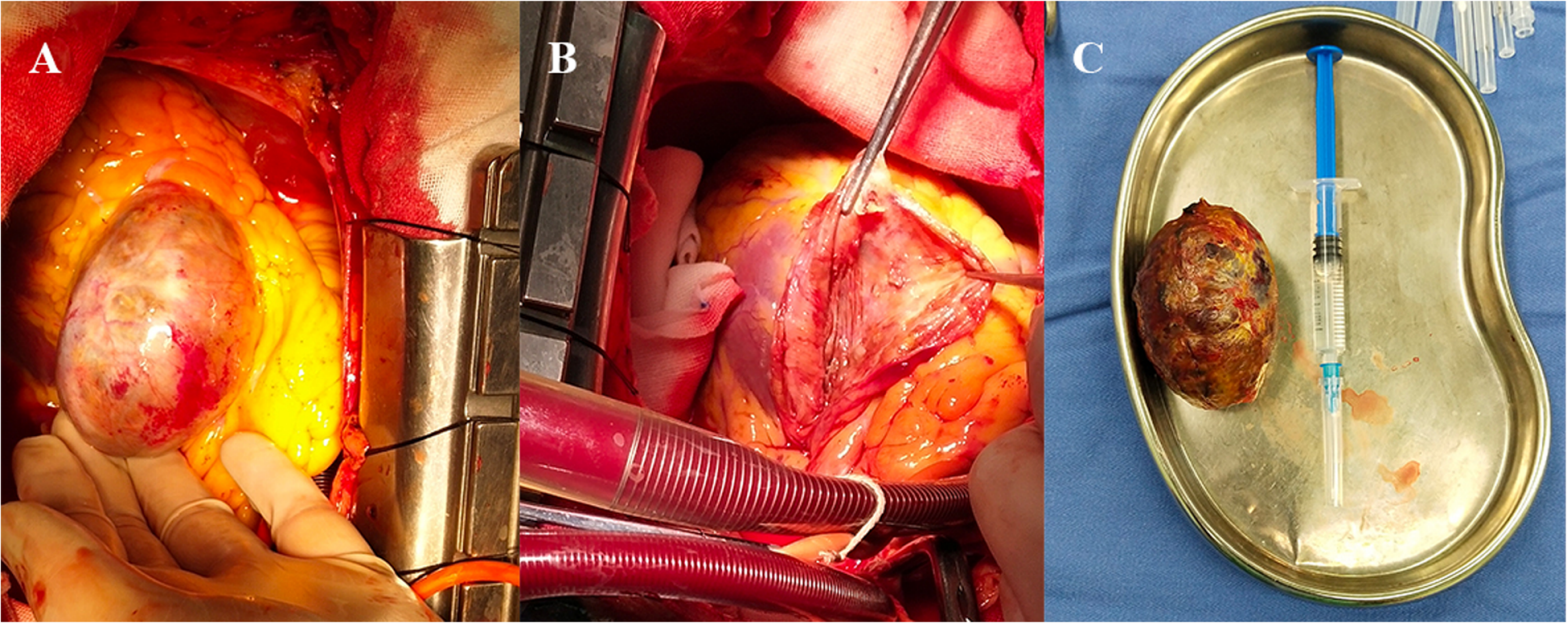
Operative photographs showing the giant aneurysm in the left anterior descending artery, a view of the inside of the aneurysmal sac and the resected aneurysm

Case Presentation: A 49-year-old male patient presented with intermittent chest distress and palpitations without an apparent cause and no chest pain or significant MI, and he was admitted to our center. The physical examination and hematological analysis showed no positive findings. Electrocardiography (ECG) showed no significant dynamic changes. Echocardiography showed a normal functioning heart (ejection fraction: 66%) with no abnormal wall motion but an “undefined spherical mass” behind the pulmonary artery. Computed tomography (CT) of the chest showed a CAA in the RCA and LAD artery; the aneurysm in the LAD artery was 6.5 × 4.5 × 4.7 cm in dimension, and thrombosis and calcification foci were detected in the aneurysmal sac (Fig. 1). To further clarify the patient’s condition, multidetector CT coronary angiography was executed to assess other cardiac structures along with the coronary arteries and showed that the anterior interventricular groove was almost occupied by the aneurysm (Fig. 2). To determine the surgical strategy, coronary angiography was performed and showed a giant coronary aneurysm in the LAD artery, with complete obstruction of the RCA (Fig. 3, Videos 1, 2, 3, 4 and 5). Then, the operation was performed under cardiopulmonary bypass (CPB) via median sternotomy, and the giant aneurysm in the LAD artery was thoroughly resected. The proximal and distal sections of the LAD artery were ligated, a saphenous vein graft was grafted into the RCA using 7–0 Prolene sutures, and the first diagonal internal thoracic artery graft was sutured to the LAD artery (Fig. 4). After the surgery, the patient was discharged home with instructions to take aspirin for life and clopidogrel for 1 year; secondary prevention of coronary heart disease was also utilized.

Discussion: In adults, atherosclerosis accounts for > 90% of CAAs, whereas Kawasaki disease is responsible for most cases in children. Recently, there have been an increasing number of cases suggesting that drug-eluting stents are related to the formation of aneurysms. CAAs are rare, and giant CAAs (greater than 5 cm) are even more uncommon [6].

The pathophysiology of CAAs is still unclear but is perceived to be identical to destruction of the arterial media; the arterial wall thins, and the wall stress increases, leading to progressive dilatation of the coronary artery segment [7]. Several hypotheses, especially for aneurysms occurring after drug-eluting stent implantation, may be postulated: extensive acute vessel damage during the initial procedure, hypersensitivity reactions, infectious processes, and phenomena resembling extreme cases of late acquired malapposition may all be implicated. Early CAAs development relating to drug-eluting stent may be a consequence of mechanical problems resulting from complicated procedures, large dissections, contained perforations, or even vessel ruptures, pseudoaneurysms may actually develop rather than true CAAS. Localized hypersensitivity vasculitis, with accumula- tion of T-lymphocytes and intense eosinophilia, was found in patients who eventually died of late DES thrombosis. Lack of endothelial coverage and severe DES malapposition caused by aneurysmal vessel enlargement was shown. Hypersensitivity reaction was thought to be caused by the polymer [8].

No distinctive clinical features of CAAs have been described. Chest pain in patients with coronary aneurysms suggestive of stable angina is the most common symptom. ST-elevation MI, non-ST-elevation MI, and sudden cardiac death may occur in these patients, while other complications, such as thrombus formation, embolization, fistula formation, rupture, hemopericardium, tamponade, compression of surrounding structures, or congestive cardiac failure, could also occur [9].

The techniques for diagnosing CAAs include noninvasive and invasive methods, such as echocardiography, CT, magnetic resonance imaging, and coronary angiography [10]. To obtain information regarding the shape, size, and location of the aneurysm, as well as coexisting anomalies, such as coronary artery disease, coronary angiography is the gold standard diagnostic tool; this method is also useful for determining the strategy for surgical resection [11].

There have been no reported controlled trials to determine the best therapy for CAAs because of the rare occurrence. Treatment selection for CAAs is mostly dependent on examining published cases and achieving an expert consensus. CAAs can be treated by medical therapy, percutaneous coronary intervention (PCI) and surgical intervention [12].

In particular, surgery is appropriate in symptomatic patients who have obstructive coronary artery disease or evidence of embolization leading to myocardial ischemia and in patients with a coronary aneurysm at risk of rupture. Various surgical strategies have been described, including resection, aneurysm ligation, marsupialization with interposition grafting, and coronary artery bypass surgery. The bulk of experience regarding these strategies stems from atherosclerosis-induced CAAs [13].

In symptomatic patients unsuitable for PCI, surgical excision or ligation of the CAA combined with bypass grafting of the affected coronary arteries is the preferred option [14]. Surgical treatment is considered to be safer and more reliable for the repair of a CAA or pseudoaneurysm. The general indications for the surgical treatment of a CAA are as follows: severe coronary artery disease; a CAA near the bifurcation of large branches; evidence of emboli from the aneurysm to the distal coronary bed resulting in myocardial ischemia; progressive CAA enlargement documented by serial angiographic measurements; a CAA in the left main stem coronary artery; complications such as fistula formation; compression of cardiac chambers; and a giant CAA (dilatation exceeding the reference vessel diameter by > four times) [12].

The prognosis of CAAs depends on the size of the aneurysm. Compared with small aneurysms, which often have a favorable prognosis and a low risk of cardiovascular events and/or mortality, giant CAAs (internal diameter > 8 mm) have a high risk of morbidity and mortality. Approximately one-half of giant aneurysms become obstructed, leading to MI, arrhythmia, or sudden death [12].

Conclusion: The main original cause of CAAs is atherosclerosis, but CAAs can also be congenital and secondary to inflammatory or connective tissue diseases (such as Kawasaki disease). The precise pathophysiology of CAAs is still unclear. While the optimal treatment for CAAs is also debatable, surgical intervention is preferred for giant CAAs.

## Supplementary information

**Additional file 1.**

**Additional file 2.**

**Additional file 3.**

**Additional file 4.**

**Additional file 5.**

## Data Availability

All data in the current report are not publicly available due to personal privacy but are available from the corresponding author upon reasonable request.

## Abbreviations

CAA: Coronary artery aneurysm
MI: Myocardial infarction
RCA: Right coronary artery
LAD: Left anterior descending
LCX: Left circumflex
CT: Computed tomography
CPB: Cardiopulmonary bypass
